# An In Vitro Engineered Osteochondral Model as Tool to Study Osteoarthritis Environment

**DOI:** 10.1002/adhm.202202030

**Published:** 2022-10-27

**Authors:** Annachiara Scalzone, Giorgia Cerqueni, Xiao‐Nong Wang, Ana Ferreira‐Duarte, Kenny Dalgarno, Monica Mattioli‐Belmonte, Piergiorgio Gentile

**Affiliations:** ^1^ School of Engineering Newcastle University Newcastle upon Tyne NE1 7RU UK; ^2^ Department of Clinical and Molecular Sciences (DISCLIMO) Università Politecnica delle Marche Ancona 60126 Italy; ^3^ Translational and Clinical Research Institute Newcastle University Newcastle upon Tyne NE1 7RU UK

**Keywords:** chondroitin sulfate dopamine, gellan gum methacrylate, in vitro models, osteoarthritis, osteochondral unit, proinflammatory mediators

## Abstract

Osteoarthritis (OA) is a joint degenerative pathology characterized by mechanical and inflammatory damages affecting synovium, articular cartilage (AC), and subchondral bone (SB). Several in vitro, in vivo, and ex vivo models are developed to study OA, but to date the identification of specific pharmacological targets seems to be hindered by the lack of models with predictive capabilities. This study reports the development of a biomimetic in vitro model of AC and SB interface. Gellan gum methacrylated and chondroitin sulfate/dopamine hydrogels are used for the AC portion, whereas polylactic acid functionalized with gelatin and nanohydroxyapatite for the SB. The physiological behavior of immortalized stem cells (Y201s) and Y201s differentiated in chondrocytes (Y201‐Cs), respectively, for the SB and AC, is demonstrated over 21 days of culture in vitro in healthy and pathological conditions, whilst modeling the onset of cytokines‐induced OA. The key metrics are: lower glycosaminoglycans production and increased calcification given by a higher Collagen X content, in the AC deep layer; higher expression of pro‐angiogenic factor (*vegf*) and decreased expression of osteogenic markers (*coll1, spp1, runx2*) in the SB. This novel approach provides a new tool for studying the development and progression of OA.

## Graphical abstract

Design and manufacturing of a highly reproducible, easy‐to‐manipulate, and low‐cost 3D in vitro osteochondral model mimicking the interface between the deep layer of articular cartilage and subchondral bone, with the aim of studying the main features of cells in these two compartments during osteoarthritis onset, compared to healthy conditions.

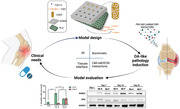

## 1 Introduction

Osteoarthritis (OA) is a degenerative disease affecting diarthrodial joints, whose causes are still unidentified. Articular cartilage (AC) degradation is a common symptom of OA development, and it causes dysfunction of the affected joint.^[^ ^1^ ^]^ In healthy AC, chondrocytes produce and maintain an abundant extracellular matrix (ECM), mainly composed of collagen type II (Coll II) and aggrecan. An imbalance between chondrocytes’ synthetic activity (anabolism) and ECM degradation (catabolism) within the AC tissue can cause a significant loss of cartilage matrix during the OA progression, associated with subchondral bone (SB) remodeling and synovial inflammation.^[^ ^2^ ^]^ Furthermore, the complex interplay between the different components of the joint makes it difficult to dissect the degradative sequence of events involved in OA pathogenesis.^[^ ^3^, ^4^, ^5^ ^]^ Recent research suggests that secretion of pro‐inflammatory cytokines into the synovial joint leads to the activation of matrix metalloproteinases (MMPs) which are responsible for the fragmentation and degradation of AC matrix, leading to bone remodeling and synovitis.^[^ ^6^ ^]^

Due to the limited self‐repair and related lack of vascularization in AC, tissue regeneration is hampered, and OA current treatments are devoted to relieving symptoms until the joint is replaced by surgery.^[^ ^7^ ^]^ The main therapies focus on symptoms management: systemic drugs are used to treat pain and inflammation, including oral analgesics and nonsteroidal anti‐inflammatory drugs (FANS).^[^ ^7^, ^8^, ^9^ ^]^ The intra‐articular injection of drugs, by targeting the only interested joint and letting the use of higher drug doses and/or a prolonged release time, is a valid alternative to systemic administration. Several systems such as hydrogels, microparticles, nanoparticles, and micelles have been developed to improve drug delivery into the joints.^[^ ^10^, ^11^, ^12^ ^]^ However, their efficiency needs to be tested both by monitoring drug release and targeting selectivity.^[^ ^13^ ^]^

In this regard, the development of in vitro models of OA meets this contingency and could be fundamental for: i) studying the disease progression from early stages, and ii) having reliable platforms to test novel drug delivery systems for the treatment of OA. Several 2D in vitro models have been exploited, both using monoculture and co‐culture approaches.^[^ ^14^, ^15^ ^]^ However, these models show drawbacks: the loss of cell phenotype when cultured in 2D, difficulties in co‐culturing different cytotypes (e.g., chondrocytes and synovial cells) using the same culture conditions, and a lack of ECM stimuli.^[^ ^16^ ^]^ Also, explants have been used as in vitro models, but their low availability and cell death at the tissue cut edges are noticeable disadvantages.^[^ ^17^ ^]^

The development of 3D biomimetic models, providing an environment emulating the native one and giving cells with appropriate structure and force, allows to study cell–cell and cell–ECM interactions, overcoming the limitation of 2D models. The homeostasis of both AC and SB tissues is influenced by biological factors and signaling molecules able to cross the demarcation zone between the tissues. As a consequence, both tissues are actively involved in the onset and progression of OA and their mutual alterations are closely related to pain and amplification of inflammation.^[^ ^18^ ^]^ Currently, a variety of in vitro OA models are available, in which the OA‐like processes are induced either mechanically—by static or dynamic compression—to emulate the tissue damage, or chemically—by culture medium supplementation with cytokines—to obtain the source of inflammatory stimuli given in vivo by synovium.^[^ ^18^ ^]^ However, none has accurately replicated all the aspects of OA, including the vital interaction between AC and SB injury/degeneration, as well as synovial inflammation.^[^ ^16^ ^]^

In this work, we developed a highly reproducible, easy to manipulate and low‐cost 3D in vitro osteochondral (OC) model mimicking the interface between the deep layer (DL) of AC and SB, with the aim of studying the main features of cells in these two compartments in healthy and OA conditions. The model design tried to reproduce the histological characteristics of the two OC regions^[^ ^19^ ^]^: for the DL, it was developed a construct with good load resistance property and porosity, made of columns able to host chondrocytes, like in the native tissue;^[^ ^20^ ^]^ for the SB, the characteristic organization of the trabecular bone was replicated.^[^ ^21^ ^]^ The choice of materials was guided by their biomimetic ability. In this regard, for the DL, we chose two natural‐based hydrogel formulations: chondroitin sulfate‐dopamine (CSDP) and gellan gum methacrylate (GGMA). CS is an important GAG present in the native AC, while GG is a natural polymer, which has recently gained attention in tissue engineering thanks to its temperature‐responsive gelation property and good mechanical properties. Both hydrogels were extensively characterized in our previous works and herein these were combined to obtain good mechanical properties since the DL of AC is characterized by the highest stiffness, given by the GGMA and good biomimicry, given by the CSDP. Furthermore, polylactic acid (PLA) synthetic polymer, which has already been widely used for bone, was selected for the SB. Due to its high hydrophobicity, it was functionalized with gelatin (GEL) to promote cell adhesion and nanohydroxyapatite (nHA) to provide biomimetic ability.^[^ ^21^, ^22^ ^]^

OA‐like changes were induced through the addition of interleukin (IL)‐1*β*, IL‐6, and tumor necrosis factor (TNF)‐*α*, the main cytokines involved in OA pathogenesis. The developed engineered tissue could provide a reliable in vitro model for proof‐of‐concept and a novel tool for testing new therapeutic options for the management of OA disease. We used a tissue engineering strategy to obtain a 3D structure respecting the histological characteristics of these regions (i.e., AC and SB) in terms of ECM composition and organization, as well as the arrangement of cells. Manufacturing techniques appropriate to the materials, soft lithography and fusion deposition modeling (FDM), were optimized to deliver the required structure. Biological assessments of both healthy and pathological DL and SB models were performed incorporating immortalized human mesenchymal stem cells (Y201s).

## 2 Results

### 2.1 Biomaterials Characterization

**Figure 1:**
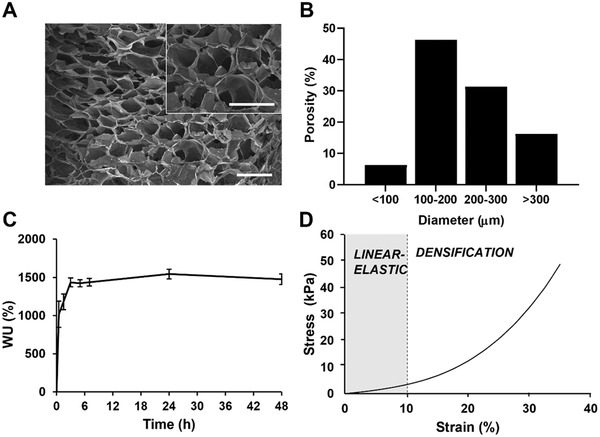
GG hydrogel characterization. A) SEM images obtained at 35× and 100× magnification; Scale bar: 500 µm. B) Distribution of pore dimensions within the range 0–500 µm, C) water uptake analysis in PBS, and D) stress–strain curve obtained from unconfined compression test, with the two main regions highlighted: linear‐elastic behavior and densification.

GGMA (3% w/v) morphology was analyzed by scanning electron microscope (SEM). Freeze‐dried cross‐section images showed a spongy morphology with open macropores, elevated interconnectivity, and anisotropic porosity (**Figure** **1**A). The analysis of pores distribution showed that ≈6% of pores have a diameter smaller than 100 µm and ≈16% bigger than 300 µm, while ≈46% of the pore dimension is in the range of 100–200 µm and ≈31% is between 200 and 300 µm (Figure 1B).

GGMA showed rapid water uptake (WU), reaching the 1016 ± 170% of WU within the first 30 min of soaking in PBS solution. This WU value gradually increased to 1433 ± 57% after 3 h and remained constant at this value up to 48 h (Figure 1C).

The stress–strain curve obtained by the static unconfined compression test showed a linear‐elastic region up to 10% of strain (Figure 1D, in gray), followed by a densification region up to 40% of strain, which represented the limit where the sample breaks. The recorded Young's modulus was 31.4 ± 4.2 kPa, calculated as the slope of the linear region of the curve (0–10% strain).

**Figure 2:**
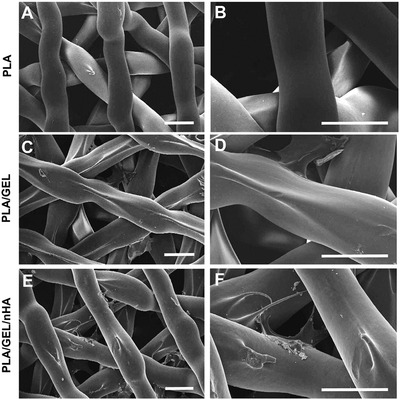
SEM analysis of PLA morphology before and after the functionalization. A,B) PLA, C,D) PLA coated with 1% w/v GEL via polydopamine coating; E,F) PLA coated with 1% w/v GEL and 5% w/w nHA via poly‐dopamine coating. Scale bar: 500 µm. Magnification: 35× (A,C,E) and 100× (B,D,F).

A 3D grid of PLA with high interconnected porosity was obtained via FDM technology. PLA grids were then functionalized with Gel and nHA, via dopamine coating. The extruded PLA filament showed a smooth and uniform surface (**Figure** **2**A,B). Following the immersion in dopamine and functionalization with GEL, the filament showed crest‐like structures on its surface (Figure 2C,D). However, no macrostructural variations were observed, as demonstrated by analysis of the filament diameter dimension (332 ± 56 µm in PLA and 378 ± 46 µm in PLA/GEL). Also, nHA addition to the scaffold (PLA/GEL/nHA) leads to uniform roughness formation, without changes in the 3D porous structure (Figure 2E,F).

### 2.2 SB Biological Assessment

**Figure 3:**
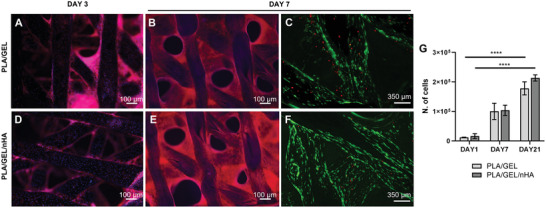
Y201 proliferation on PLA/GEL and PLA/GEL/nHA 3D printed scaffolds. Staining of cells’ cytoskeleton (Phalloidin‐Rhodamine) and nuclei (DAPI) on PLA/GEL at A) day 3 and B) day 7; and on PLA/GEL/nHA at D) day 3 and E) day 7. Live and dead staining on C) PLA/GEL and on F) PLA/GEL/nHA at day 7. G) MTT assay at days 1, 7, and 21 on PLA/GEL and PLA/GEL/nHA, showing the number of Y201 cells at each time point. Statistics: ****p < 0.0001.

To investigate the effect of PLA functionalization with Gelatin and nHA on cells adhesion and viability, Live/Dead staining was performed after 3 days of culture for each step of the process: i) PLA coated with dopamine (PLA/DOPA); ii) PLA coated with dopamine and functionalized with gelatine (PLA/GEL); and iii) PLA/GEL with the addition of nHA (PLA/GEL/nHA). A significant increase in the number of live cells was detected on scaffolds functionalized with gelatine compared to the scaffolds with only dopamine, where more dead cells were qualitatively observed (Figure S1D, Supporting Information). No differences were detected in terms of cell spreading and adhesion in the three conditions (Figure S1A–C, Supporting Information). Since gelatin‐functionalized scaffolds showed better cell viability, PLA/DOPA was preferred for the following experiments. Y201s maintained their viability on PLA/GEL and PLA/GEL/nHA scaffold for up to 7 days and just a few dead cells were observed (**Figure** **3**C,F). Cells were homogenously distributed and spread on PLA/GEL and PLA/GEL/nHA scaffold filaments at day 3 (Figure 3A,D), whereas they entirely covered the scaffold at day 7 (Figure 3B,E). This proliferative tendency was assessed via Thiazolyl Blue Tetrazolium Bromide (MTT) assay on the scaffold with and without the incorporation of nHA, up to 21 days of culture (Figure 3E): cells number increased over 21 days of culture (*p* < 0.0001) and approximately doubled from day 7 to day 21 (*p* < 0.0001) onto both PLA/GEL and PLA/GEL/nHA.

### 2.3 DL Biological Assessment

**Figure 4:**
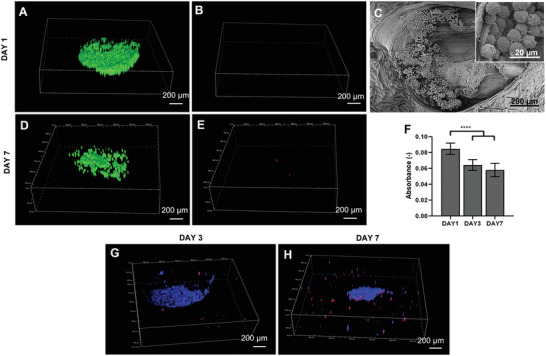
Y201‐Cs viability when embedded within GGMA/CS hydrogel. Live/Dead viability assessment of 3D construct at A,B) day 1 and D,E) day 7, respectively, A,D) live and B,E) dead. SEM images at C) day 1: 250× and 2500× (inset). F) Histogram of cell viability obtained by MTS assay on days 1, 3, and 7. DAPI/Phalloidin staining at G) day 3 and H) day 7. Statistics: ***p < 0.001.

Chondrocyte viability when embedded in GGMA/CS was assessed via Live/Dead. Viable cells agglomerated within the GGMA channels were observed both on days 1 and 7 (**Figure** **4**A,B,D,E), with a few dead cells observed on day 7 (Figure 4E). Cell distribution within the CS in the GGMA channel was also displayed by DAPI and phalloidin staining (Figure 4C,F). On day 7, Y201‐Cs partially lost the initial assemblage provided by the seeding (Figure 4D,F): most of the cells were stacked one on top of the other inside the channels, while some of them diffused within GGMA hydrogel. GGCS hydrogels were able to sustain the round chondrocyte morphology, as shown by SEM images (Figure 4G,H). Y201‐Cs metabolic activity was analyzed via CellTiter 96 MTS (3‐[4,5,dimethylthiazol‐2‐yl]‐5‐[3‐carboxymethoxy‐phenyl]‐2‐[4‐sulfophenyl]‐2H‐tetrazolium) assay and showed a slight decrease on days 3 and 7, with respect to day 1 (Figure 4I).

### 2.4 Y201s Osteogenic Commitment onto SB

**Figure 5:**
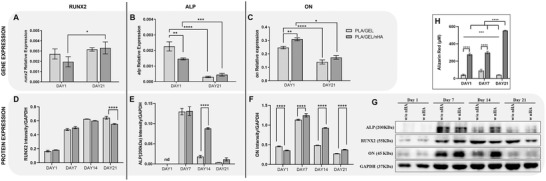
Gene (via RT‐qPCR), protein (via Western Blot) expression, and calcium deposition (Alizarin Red) analyses to evaluate the ability of functionalization to promote Y201s osteogenic differentiation onto PLA/GEL scaffold with (PLA/GEL/nHA) and without the addition of nHA (PLA/GEL). A–C) Gene relative expression of A) *runx2*, B) *alpl*, and C) *on* by Y201s via RT‐qPCR on day 1 and day 21 of culture. Proteins densitometric quantification of D) RUNX2, E) ALP active peptide 200 kDa and F) ON expressed as intensity normalized to GAPDH on days 1, 7, 14, and 21. G) Western blotting membrane of Y201s on days 1, 7, 14, and 21 incubated with ALP, RUNX2, ON, and GAPDH antibodies. H) Alizarin Red quantification at days 1, 7, and 21. Statistics: *p < 0.05, **p < 0.01, ***p < 0.001, ***p < 0.0001.

The intrinsic ability of nHA‐functionalized PLA to support Y201 osteogenic commitment was investigated by gene and protein expression analysis as well as by quantification of calcium extracellular deposition. The *runx2* mRNA content was stable over time and between samples (**Figure** **5**A), showing just a slight increase (*p* < 0.05) at day 21 in PLA/GEL/nHA samples, while the protein amount increased in a time‐dependent manner (Figure 5D,G). *alpl* mRNA was expressed from day 1 and then downregulated at day 21 in both samples (*p* < 0.001 for PLA/GEL/nHA and *p* < 0.0001 for PLA/GEL) (Figure 5B). The highest expression of ALP active enzyme (200 kDa peptide) appeared on day 7 for both samples and was preserved high in PLA/GEL/nHA scaffolds up to 14 days (Figure 5E,G). Osteonectin (ON) was detected from day 1, reached a peak on day 7 and then gradually decreased showing the same trend of mRNA on day 21 (Figure 5C,F). Interestingly, ON gene and protein expressions were upregulated in PLA/GEL/nHA scaffolds at each time point (*p* < 0.0001) (Figure 5C,F,G). Furthermore, analysis of the extracellular calcium deposition showed that scaffolds functionalized with nHA were able to encourage mineralization at each time point (*p* < 0.0001), in comparison with the not‐functionalized one, with a notable increase after 21 days of culture (Figure 5H) (*p* < 0.0001).

### 2.5 Assessment of Pathological Features in the OC Model

**Figure 6:**
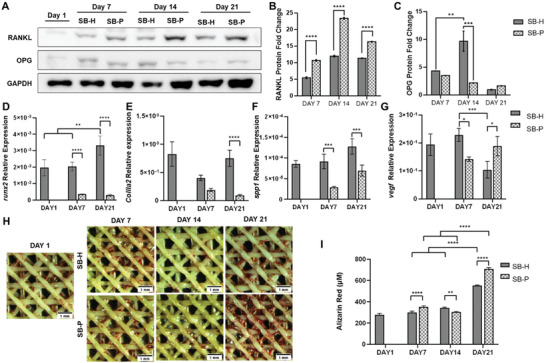
Assessment of SB features in H and P conditions. A) Western blotting membrane incubated for RANKL, OPG, and GAPDH at days 1, 7, 14, and 21. Histograms depicting densitometric quantitation of western blotting for B) RANKL and C) OPG. Normalized results were expressed as intensity at days 7, 14, and 21 with respect to day 1. Relative gene expression of D) *runx2*, E) *collIa2*, F) *spp1*, and G) *vegf* on days 1, 7, and 21 in H and P conditions. H,I) Alizarin red analysis on days 1, 7, 14, and 21 in H and P conditions, both qualitative and qualitative. Statistics: *p < 0.05, **p < 0.01, ***p < 0.001, and ****p < 0.0001.

The SB zone behaviors in healthy (SB‐H) and pathological (SB‐P) conditions were compared in terms of ability to trigger bone remodeling, production of factors involved in ECM production and ECM mineralization. In SB‐P samples, RANKL expression was significantly upregulated at each time point (*p* < 0.0001) (**Figure** **6**A,B) while OPG production was maintained low and stable. At day 14, OPG was significantly upregulated in SB‐H (*p* < 0.001) (Figure 6A,C).

Analysis of gene expression of *runx2* (Figure 6D) and *spp1* (Figure 6F) showed their significant downregulation in SB‐P at each endpoint (*p* < 0.0001), while *collIa2* reduction was significant after 21 days of culture (*p* < 0.0001) (Figure 6E). At 7 days of culture, *vegf* showed its highest expression in SB‐H, though after 21 days its expression decreased in H samples and increased in P ones (*p* < 0.05) (Figure 6G).

The Alizarin red qualitative staining confirmed an increase of extracellular mineral deposition at day 21 mainly in zones of PLA fibers intersection (Figure 6H), with a significantly higher rate in SB‐P (*p* < 0.0001) (**Figure** 6I).

**Figure 7:**
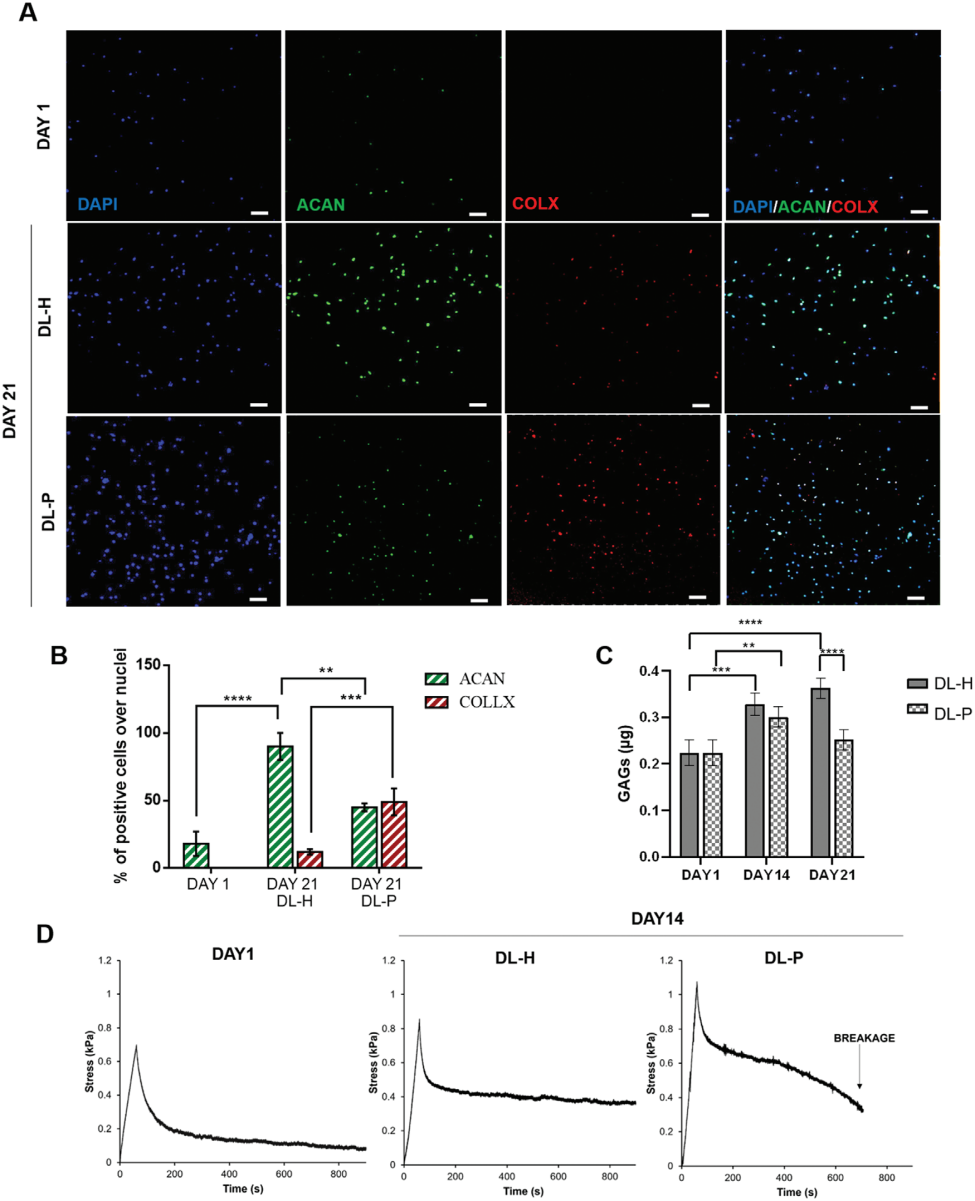
Assessment of pathological features on DP side (GGMA/CS) by comparing healthy (DP‐H) and pathological model (DP‐P). A) Immunofluorescence staining for ACAN (green) and COLLX (red) at days 1 and 21 for DL‐H and DL‐P: cell nuclei were stained with DAPI. Scale bar: 100 µm. B) The histogram shows the percentage of ACAN and COLLX positive cells with respect to the nuclei. C) Quantification of GAGs produced by chondrocytes at day 1, 14, and 21 in H and P conditions; acellular GGMA/CSDP background was subtracted, and data extrapolated from a standard curve. D) Stress and relaxation curve of AC construct with embedded chondrocytes at day 1 and 14, in H and P conditions. Statistics: *p < 0.05, **p < 0.01, ***p < 0.001, and ****p < 0.0001.

The behavior of the DL in a healthy (DL‐H) and pathological (DL‐P) environment was investigated evaluating the expression of cartilage specific markers and monitoring the DL viscoelastic properties. GGMA/CS hydrogel 3D environment was able to sustain Y201‐Cs round morphology up to 21 days in both conditions (**Figure** **7**A). Figure 7A,B shows Aggrecan (ACAN) and Collagen X (COLLX) relative intensities qualitatively and quantitatively: the expression of ACAN, by embedded chondrocytes, was very low at day 1 and abundantly upregulated after 21 days of culture in DL‐H environment. Contrarily, COLLX was undetected on day 1 in both samples, while it was observed in a few cells at day 21 in DL‐H model and in a great number of cells in DL‐P model (ACAN: day 1 18 ± 9, day 21 DL‐H 90 ± 10, day 21 DL‐P 45 ± 3; COLLX: day 1 n.d.; day 21 DL‐H 12 ± 2, day 21 DL‐P 49 ± 10). The merged images of DAPI, ACAN, and COLLX stains showed that the pattern of expression was inhomogeneous: some cells were stained only for COLLX or ACAN, others for both, while few of them for none of the proteins (Figure 7A). Moreover, chondrocyte GAGs production increased with time (*p* < 0.0001) in DL‐H, while it showed a decrease at day 14 and day 21 in DL‐P samples (Figure 7C). Stress–relaxation graphs show the change in behavior of the DL‐emulating hydrogel over 14 d of culture. Both on days 1 and 14, *σ*/*t* in the DL showed a typical curve of a viscoelastic material, with a difference in the peak modulus (*E*_P_) (7.8 ± 0.1 kPa on day 1 vs 10.7 ± 0.3 kPa on day 14 in DL‐H) and the equilibrium modulus (*E*_Y_) (1.1 ± 0.2 kPa at day 1 vs 4.1 ± 0.2 kPa at day 14 in DL‐H). Also, the relaxation times obtained on day 1 were: viscoelastic relaxation time (*τ*_1_) 12.8 ± 1.3 s and poroelastic relaxation time (*τ*_3_) 2698.8 ± 727.6 s. On day 14, DL‐H model showed values of *τ*_1_ and *τ*_3_, respectively, 7.7 ± 2.0 and 1471.0 ± 191.6 s. The pathological model tested at 14 days (DL‐P) showed an atypical *σ*/*t* curve, because samples never reached the equilibrium and the curve suddenly fell after 400 s. In this case, the calculated *E*_P_ was 12.1 ± 0.1 kPa, *τ*_1_ 8.8 ± 2.4 s, and *τ*_3_ 1693.5 ± 634.3 s. The value of *τ*_3_ calculated does not take into consideration the breaking of the sample and, in this regard, it was not possible to calculate *E*_Y_, as the sample never reached the equilibrium.

## 3 Discussion

The OC interface is a functioning synergistic unit, with a close physical association between SB and AC, suggesting the existence of biochemical and molecular crosstalk across the OA interface in healthy and pathological conditions.^[^ ^23^ ^]^ In vitro reproduction of this complex should functionally and structurally mimic the native OC tissue and provide an appropriate environment sustaining cell viability and ECM production and maintenance.^[^ ^24^ ^]^

GGMA and CSDP were previously characterized and selected at concentration of 15% w/v for CSDP and 3% w/v for GGMA. GGMA 3% possessed Young's modulus of 31.4 ± 4.2 kPa, which falls within the range of hydrogels used for AC, good hydrophilicity, and a high and well‐distributed porosity, with a pore size mainly in the range 100–300 µm, optimal for nutrient exchange. After the materials setup optimization, the SB‐DL model was evaluated in healthy and pathological (cytokines‐induced) environments. Cytocompatibility studies confirmed the biomaterials’ ability to sustain and promote cell activity. Y201‐Cs within the DL showed the physiological round chondrocytes phenotype, and Y201s showed an elongated morphology.^[^ ^24^, ^25^ ^]^ Also, the PLA functionalization with GEL sustained cell viability and proliferation, and functionalization with nHA did not negatively affect these phenomena.

The ability of PLA functionalization with nHA to support Y201s osteogenic commitment was also investigated. The mRNA content of *runx2*, a transcription factor required to trigger and sustain the osteoblasts differentiation, was stable during cell culture and between samples; its protein expression increased with time to meet the OBs maturation needs.^[^ ^26^ ^]^ ALP is one of the early osteoblasts differentiation markers and participates in ECM mineralization.^[^ ^27^ ^]^ In accordance with their ability to undertake the osteogenic lineage, Y201s expressed elevated *alp* mRNA from day 1 while the expression of the active enzyme was upregulated on day 7. Functionalization with nHA sustained ALP expression as also demonstrated by other authors.^[^ ^28^ ^]^ Thus, the ALP expression on the PLA scaffold was in line with the osteogenic differentiation steps and we can speculate that nHA improved ALP production improving cells' mineralization potential. ON is one of the most abundant noncollagenous proteins expressed during the late stage of osteoblasts differentiation by mature OBs.^[^ ^29^ ^]^ Its expression is related to the initial formation of mineral bone.^[^ ^30^ ^]^ Cells on PLA/GEL/nHA showed a higher expression of ON in terms of mRNA and mature protein contents over all the culture period, confirming its potential to assist bone formation. In conclusion, PLA/GEL/nHA samples showed an enhanced expression of osteoblast related markers compared to PLA/GEL; therefore, nHA functionalized scaffold was selected for the following experiments.

Contrary to the common description of OA as “wear and tear” pathology, proinflammatory mechanisms can initiate and participate in the tissue modifications related to the pathology progression, via pro‐inflammatory cytokines.^[^ ^31^ ^]^ In this study, IL‐6, TNF‐*α*, and IL‐1*β* were chosen according to their prevalence in the OA synovial fluid.^[^ ^32^ ^]^ In the SB, the presence of inflammatory cytokines altered Y201s behavior. Particularly, RANKL expression (a factor involved in osteoclasts' recruitment and function) increased exponentially, showing the potential of the model to promote resorption activity and to mimic the remodeling rate characterizing osteoarthritic SB, that appears in the early phase of the disease.^[^ ^33^ ^]^ In support of these data, the expression of OPG (a molecule that protects bone from excessive resorption by binding to RANKL) was inversely related to RANKL expression, especially at day 14. Data detected at day 21 suggested a different balance of the remodeling activity, which needs further investigation. VEGF is a marker related to pathology progression and pain;^[^ ^34^, ^35^ ^]^ its expression increases during the late stage of OA. In our model, the *vegf* gene expression at SB level was initially downregulated (day 7) and then upregulated in the pathological environment. It must be highlighted that our model, at the SB level, seems to recapitulate the timing of OA pathology progression, with an early phase (day 7) characterized by a strong remodeling capability and a late phase (day 21) showing a decrease of the remodeling ability to give space to the vascularization phenomenon. Furthermore, *runx2* gene expression, which is the main activator of osteogenic commitment, was downregulated in the pathological environment with a consequential decrease of downstream osteogenic markers.^[^ ^36^, ^37^ ^]^ These data were in line with the literature that confirms the role of TNF‐*α* and IL‐1*β* in the reduction of osteoblasts differentiation through inhibition of Runx2 expression and function.^[^ ^38^, ^39^, ^40^ ^]^ On the contrary, a significant increase in extracellular calcium deposition was detected in the pathological environment, after 21 days of culture. However, the role of mineralization needs more in‐depth investigations because it could be correlated both to the thickening of the interface between AC and SB as well as to the lack of remodeling activity. Overall, our data demonstrated the capability of the model to modulate the mineralizing capacity in a healthy and pathological environment.

In the AC, inflammatory cytokines affect also ACAN (the most abundant proteoglycan in AC) synthesis and degradation, especially in the early phase of the OA.^[^ ^41^, ^42^, ^43^ ^]^ ACAN is the main responsible for the compressive loads’ resistance, thanks to the osmotic asset of its GAGs.^[^ ^44^ ^]^ The pathological environment changed Y201‐Cs’ ECM production with the alteration of GAGs synthesis and the decrease of ACAN expression coming out in the collapse of our hydrogels' viscoelastic properties. Conversely, the production of ACAN and GAGs in healthy conditions increased with the extension of culture time in harmonization with an increase of the overload resistance. During OA, AC homeostasis is also disturbed by hypertrophic chondrocyte differentiation towards apoptosis. To understand if the model was suitable to promote chondrocyte hypertrophy in a pathological environment, the expression of collagen X, which is an important marker for AC calcification, was investigated.^[^ ^45^ ^]^ Its expression was very low in DL‐H models, while greatly upregulated in the pathological situation, thus giving its contribution to the ECM weakness and the establishment of OA features.

## 4 Conclusions

A highly reproducible 3D in vitro model of the osteochondral unit has been developed, endowed with quick manufacturing, easy manipulation, and easy material availability. The main feature of this model was its unique design that mimics the in vivo architecture of the AC and SB. The design was carried out 1) by analyzing the histological and structural features of the two tissues of interest and 2) by choosing biomaterials capable of respecting the designed characteristics and the biological and structural parameters of the tissues. The in vitro model was able to create an effective environment for physiological cell behavior, sustaining the expression of appropriate tissue‐related markers. In addition, it provides a tool for studying cell fate during OA onset, as well as a new platform to further test new therapeutics or to study crosstalk interactions and molecular pathways.

## 5 Experimental Section

### Materials

All the materials were obtained from Sigma Aldrich UK, unless otherwise stated.

### Biomaterials Preparation as Building Blocks for the Osteochondral Model

GGMA Synthesis, Hydrogel Preparation, and Characterization: GG hydrogel was produced as previously reported.^[^ ^46^ ^]^ Briefly, highly methacrylated GGMA was synthesized by a reaction between Gellan Gum (Gelrite, Molecular weight [MW] = 1.000.000 g mol^−1^) and methacrylic anhydride (MA), by dissolving 1% w/v of GG in TRIS 1 m (Trizma base) at pH 8.5–9.0 for 30 min at 90 °C, before adding MA (8% w/v) to the GG solution. The reaction was kept at 50 °C for 5 h, and the obtained GGMA formulation was purified for 3 days by dialysis against distilled water (dH_2_O), using cellulose membrane (MW cut‐off of 11–14 kDa). The dialyzed GGMA was lyophilized for 48 h in a freeze‐dryer (Alpha 1‐2 LDplus, CHRIST, Germany). Hydrogels were prepared from freeze‐dried GGMA in dH_2_O (3% w/v). To perform the physicochemical analysis, the hydrogel solution was poured into a 48‐well plate (500 µL per well) and left crosslink under UV at 254 nm for 5 min.

The morphology of the freeze‐dried gels (after‐gelation) was assessed using JEOL JSM‐5600LV scanning electron microscope. Samples were gold‐coated using a BIO‐RAD Sputter Coater machine. Their morphology was observed and recorded at 6 mm working distance, 20 kV operation voltage, and two different magnifications (35×, 100×). The obtained images were analyzed with ImageJ software, to calculate the porosity distribution of each sample (40 pores for each image and three images in total).

To analyze the water uptake kinetics of GG 3%, three freeze‐dried samples (after‐gelation) were considered. GG lyophilized gels were singularly weighted, put in a 5 mL vial with 3 mL of Dulbecco's phosphate‐buffered saline (PBS) and stored at 37 °C. The hydrogels were dried on filtered paper and weighed at each time point (30 min, 1, 3, 5, 8, 24, and 48 h of incubation). The wet weight was measured (Wt) and compared to the initial wet weight (Wi). The water uptake (WU) was defined according to Equation (1)

(1) $$\mathrm{WU}\left(\%\right)=\left(W_{t}-W_{i}\right)/W_{i}x100$$

Finally, an unconfined compression test was performed with a mechanical testing machine (EZ‐SX, 20 N load cell, Shimadzu, Japan) to analyze the mechanical properties of the produced hydrogels. Three samples were compressed at a rate of 1 mm min^−1^ during the hydrogel rupture. Young's modulus (*E*) was derived from the stress/strain graph (slope of the linear‐elastic area of the curve [0–10% strain]).

CSDP Synthesis and Hydrogel Preparation: CSDP was synthesized, as previously reported by Scalzone et al.^[^ ^47^ ^]^ Briefly, chondroitin 4‐sulfate sodium salt from bovine trachea (CS; MW = 515.376 g mol^−1^) (10% w/v) was dissolved in a buffer (pH 6) of 0.1 m 2‐(N‐Morpholino) ethane sulfonic acid (MES; MW = 195.24 g mol^−1^) and 0.5 m sodium chloride (NaCl; MW = 58.44 g mol^−1^). Then, at a molar ratio of 1:1:1, CS,1‐ethyl‐3‐(3‐dimethylaminopropyl)‐carbodiimide hydrochloride (EDC; MW = 191.70 g mol^−1^) and N‐hydroxy succinimide (NHS; MW = 115.09 g mol^−1^) were mixed for 30 min under stirring. The CS/EDC/NHS solution was added to dopamine hydrochloride (MW = 189.64 g mol^−1^) and stirred for 4 h at pH 6.0. The dialysis and lyophilization were then carried out as described previously for the GGMA, with the addition of an acidic wash (acidified dH_2_O at pH < 2) on the last day. CSDP hydrogels were obtained by the crosslinking of catechol groups. Freeze‐dried CS/DP was dissolved at 15% w/v in cell media (DMEM/F12). Sodium periodate (NaIO_4_) was added to the formulation at physiological pH and the sol/gel transition happened in 30 s.

PLA 3D Printing and Functionalization: Rokit INVIVO 3D bioprinter (RokitHealthcare) was used to extrude a PLA (Ingeo Biopolymer, Nature Works) grid to simulate the SB, by using the FDM head (nozzle size 0.4 mm). The extrusion temperature was set to 215 °C, the printing and travel speed at 2 mm s^−1^, the infill pattern was a grid, and the rotation angle was set to 45° between consecutive layers. Each printed scaffold had a dimension of 13 × 13 × 2 mm. PLA scaffolds were functionalized with nHA and gelatin (Gel) via polydopamine coating as described in a previous work.^[^ ^48^ ^]^ Briefly, the scaffolds were incubated in polydopamine solution (DP dissolved in TRIS buffer 0.1 m pH 8.5) overnight and then washed in dH_2_O and incubated for 7 h in Gel (1.5% w/v) and nHA (5% w/w). SEM analysis was performed to analyze the morphology of the obtained scaffolds.

### Cell Culture Protocol

Human TERT immortalized bone marrow stromal cell line (Y201s)^[^ ^49^ ^]^ was cultured at 37 °C, 5% CO_2_, in low glucose DMEM with the addition of 10% fetal bovine serum (FBS), 2 × 10^−3^ m l‐glutamine, and a 1% penicillin–streptomycin (P/S) mixture (100 U mL^−1^). To differentiate the Y201 stem cells to chondrocytes (Y201‐Cs), cells were grown at 37 °C, 5% CO_2_ in DMEM with P/S supplemented with 1% ITS+1, 10 ng mL^−1^ TGF‐*β*3, 40 µg mL^−1^ L‐Proline, 100 × 10^−9^ m dexamethasone, 50 µg mL^−1^ l‐ascorbic acid‐2‐phosphate for 21 days.^[^ ^50^ ^]^ Differentiated cells (Y201‐C) were cultured in DMEM/F12 supplemented with 10% FBS and 1% P/S and used at passage 14 after differentiation for the model manufacturing and evaluation. For the setup of the healthy condition, cells were cultured within the manufactured OC model at 37 °C and 5% CO_2_ with the addition of DMEM/F12. The pathological condition was set up at day 1 of culture, by adding to each OA sample an optimized cytokines cocktail (IL‐1ß and TNF‐*α* at 1 ng mL^−1^, and IL‐6 at 10 ng mL^−1^).

### Osteochondral Model Manufacturing

**Figure 8:**
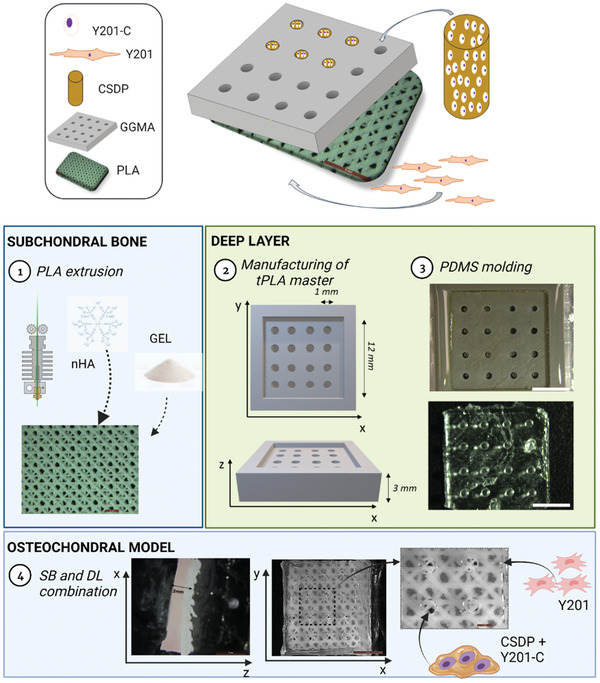
Schematic of the manufacturing of 3D in vitro model of OA, following a four‐step process: manufacturing of PLA with FDM technique and functionalization with GEL and nHA (1), manufacturing of DL via soft lithography technique (2–3), combination of SB and DL in the osteochondral model (4).

For the manufacturing of the OC construct in vitro, a multistep approach was followed, as illustrated in **Figure** **8**:

Briefly:

1. PLA was extruded and functionalized with PLA and GEL, as reported in the previous section. 3, for manufacturing the SB.
2. The soft lithography technique was implemented for the obtainment of a channeled GG construct. As a first step, it was manufactured the GG positive master by 3D printing a PLA structure (tPLA) (14 × 14 × 2 mm), provided with 16 channels (1 mm diameter each), by using an Ultimaker2 printer (Netherland). The tPLA filament (2.85 mm thick) was extruded with a 0.40 mm nozzle at a speed of 35 mm s^−1^ and infill of 100%. This structure was used for the production of a negative polydimethylsiloxane (PDMS)‐based mold, composed of multiple pins, by using SYLGARD 184 Silicone Elastomer Kit (silicon monomer solution and curing agent at volume ratio 10:1). The PDMS was let to react at room temperature (RT) for 24 h and, after that, the mold was detached from the PLA master, sterilized under UV (254 nm) and ready for the manufacturing of the DL multichanneled construct.
3. For the OC construct setup, PDMS molds were transferred into a 12‐well plate and 2 mL of GGMA were poured above each mold. The multiwell was exposed for 5 min to UV (254 nm) for the GGMA photo‐crosslinking before gently placing the PLA/GEL/nHa scaffolds on the top of each mold. The whole construct was left other 10 min under UV and, then, 500 µL of DMEM/F12 were added to each well to complete the crosslinking and the adherence of GGMA hydrogel to the PLA. After the complete crosslinking, the PDMS mold was removed, and a structure made of multichanneled GGMA adhering to the PLA scaffold was obtained. Then, in each channel of the GGMA structure, 20 µL of the pre‐crosslinked CS embedded with Y201‐Cs (3.5 × 10^6^ cells mL^−1^) were added.^[^ ^51^ ^]^ The construct was incubated at 37 °C, 5% CO_2_ to complete the chemical crosslinking for 5 min. Then, it was turned upside down in the multiwell and Y201s were seeded on the PLA at a concentration of 1:4 compared to Y201‐Cs.

Finally, the obtained constructs were incubated at 37 °C, 5% CO_2_ with the addition of DMEM/F12 and the analyses were performed over 21 days of culture. The pathological condition was set up in OA samples on day 1 of culture, by adding to each sample a mixture of cytokines to chemically induce the OA inflammation environment (IL‐1ß and TNF‐*α* at 1 ng mL^−1^, and IL‐6 at 10 ng mL^−1^).

### Biological Assessment of the In Vitro Osteochondral Model

The SB and DL components were analyzed separately by using the following assays.

Cells Viability, Immunostaining, and Metabolic Activity of the DL and SB Constructs: Cell viability was assessed with a fluorescence‐based kit (LIVE/DEAD Cell Imaging Kit, Life Technologies, UK) according to the manufacturer's instructions. For the SB, the Live/Dead assay was assessed before and after the functionalization (on bare PLA, PLA/GEL, PLA/GEL/nHA samples) on day 3 of cell culture.

Cells distribution was assessed with immunostaining assays: Samples were fixed in 4% w/v paraformaldehyde (PFA) for 30 min at 4 °C. Then, cells were permeabilized with 0.1% v/v Tween20 in PBS, and samples were incubated with phalloidin‐tetramethylrhodamine B isothiocyanate (Phalloidin‐Rhodamine) solution (2 µg mL^−1^ in 0.1% PBS/Tween20) for 30 min at RT to stain their cytoskeleton. Samples were then washed with 0.1% PBS/Tween20 solution and immersed in 4′,6‐diamidino‐2‐phenylindole (DAPI) solution (Vector Laboratories, UK) (1 µg mL^−1^ in 0.1% PBS/Tween20) for 10 min at RT to stain their nuclei.

For both Live/Dead and immunostaining analyses, images were collected on days 1 and 7, by using an EVOS M5000 microscope for the PLA and using a Nikon A1R inverted confocal microscope for the GGMA/CSDP and analyzed with NIS‐Elements Microscope Imaging Software.

The metabolic activity of Y201‐Cs encapsulated in the DL construct was evaluated with CellTiter 96 AQueous MTS Reagent (Promega) according to the manufacturer's instruction on days 1, 3, and 7, while the metabolic activity of Y201s, seeded on the SB construct, was tested using MTT assay, following the supplier's instructions, at days 1, 7, and 21 on PLA functionalized with PLA/GEL and PLA/GEL/nHA. The estimation of the cell number was performed based on a standard curve, generated by seeding Y201s at different densities (from 5 000 up to 500 000). Readings were performed with a FLUOStar Omega Plate reader (BMG LABTECH, UK).

Cell Morphology: SEM analysis (Tescan Vega LMU SEM, Tescan) was performed for PLA and GGMA/CS after 24 h of culture. Samples were fixed in 2% glutaraldehyde for 1 h at 4 °C, rinsed in 0.5 m cacodylic acid buffer and dehydrated in ethanol grades: 30 min in each 25%, 50%, 70%, 80%, twice in 95%, and four times in 100% EtOH. Samples were stored at 4 °C in 100% EtOH until the critical point dried. Finally, gels were mounted on carbon discs, gold‐coated using a Polaron E5000 SEM Coating unit (Quorum Technologies Ltd, UK) and imaged.

### Osteogenic Assessment of SB: RT‐PCR, Western Blot, and Alizarin Red

To evaluate the effect of the nHA‐functionalization on Y201 seeded on the PLA, PLA/GEL/nHA, and PLA/GEL constructs were compared in terms of cell osteogenic commitment, via gene expression analysis, protein expression analysis, and calcium deposition.

Gene Expression Analysis: For the gene expression analysis, mRNA was extracted from the samples using Qiagen miRNeasy Micro Kit (Qiagen, Germany) and transcribed to cDNA using a High‐Capacity cDNA Reverse Transcription Kit (Thermo Fisher, UK), according to the manufacturer's protocol. PCR was performed with 0.5 µg of cDNA using TaqMan Fast Advanced Master Mix for the TaqMan gene expression assay kits *runx2* (Hs01047973_m1), *alpl* (Hs01029144_m1), and *sparc* (Hs00234160_m1) and a qRT‐PCR machine (QuantStudio 3, Thermo Fisher Scientific, USA) was used as explained before.^[^ ^50^ ^]^ GAPDH (Hs99999905_m1) was used as a housekeeping gene. Gene expression was normalized to GAPDH and expressed relatively using the 2^−(ΔCt)^ method. Relative expression (RE)^[^ ^52^ ^]^ on day 1 and day 21 was reported to assess Y201s osteogenic commitment.

Western Blot: Protein expression analysis was performed as previously described.^[^ ^53^ ^]^ Total proteins were extracted from Y201s after days 1, 7, 14, and 21 of culture in healthy and OA conditions using the RIPA Lysis Buffer System (sc‐24948, Santa Cruz Biotechnology Inc.) supplemented with protease inhibitors (S8820, Sigma‐Aldrich, Italy). Briefly, scaffolds were washed with PBS and incubated with 100 µL of RIPA Lysis buffer for 30 min. The extraction was improved by pipetting the buffer every 10 min and keeping the plates on ice. Protein extracts were stored at −20 °C until use. Protein concentration was measured by DC protein assay (LIT448D, Bio‐Rad). Total protein extracts (20 µg) were incubated with Tris‐Glycine SDS Sample Buffer (2×) (Novex) according to the manufacturer's instructions, fractionated in 4%–15% SDS‐PAGE gel. Gels (HC1000 Surecast, Thermo Fisher Scientific) were electrophoretically transferred to 0.2 µm nitrocellulose membranes (Bio‐rad). Membranes were incubated with 5% milk in Tris‐buffered saline with 0.1% Tween 20 (TBS‐T) to block nonspecific sites and then with rabbit anti‐Runx2 (55 kDa, 4 µg mL^−1^, HPA022040, Sigma‐Aldrich), mouse anti‐ALP (200 kDa, dil 1:1000, ab126820, abcam), and mouse anti‐ON (45 kDa, 0.4 µg mL^−1^, Sc‐73472, Santa Cruz Biotechnology) antibodies at 4 °C. Mouse anti‐GAPDH (36 kDa; 0.1 µg mL^−1^, 60004‐1‐Ig, Proteintech) was used as an endogenous control. After overnight incubation, the membrane was washed with TBS‐T and then incubated with anti‐rabbit (0.8 µg mL^−1^, Sc‐2004, Santa Cruz Biotechnology) and anti‐mouse (0.1 µg mL^−1^, A90‐116P, Bethyl) secondary antibodies conjugated to horseradish peroxidase for 1.30 h at room temperature. The detection of antibody binding was performed with Pierce ECL Western Blotting Substrate (Thermo Scientific) and images were acquired with an Alliance Mini HD9 (Uvitec, Cambridge, UK). Densitometric analysis was performed with ImageJ software (https://imagej.nih.gov/ij/download.html).

Alizarin Red: To detect the calcium deposits, PLA/GEL and PLA/GEL/nHA samples on days 1, 7, 14, and 21 were fixed in 4% PFA, washed in PBS twice and stained with 1 mL of Alizarin Red solution (40 × 10^−3^ m in dH_2_O) for 30 min at RT. Then, samples were washed with dH_2_O and dried overnight at 50 °C in a 5% CO_2_ atmosphere. Imaging of the samples was performed with a stereomicroscope (Leica Microsystems). To quantify the Alizarin Red, 10% acetic acid was added to each stained sample and let under shaking for 30 min, the acetic acid solution was transferred to a 1.5 mL Eppendorf tube, heated to 85 °C for 10 min and then placed on ice for 5 min. Then, the solutions were treated with 10% ammonium hydroxide to neutralize the acetic pH to 4.1–4.5 and the reading was performed in duplicated at 405 nm absorbance with a FLUOStar Omega Plate reader (BMG LABTECH). The standard curve was obtained in the range of 0–2 × 10^−3^ m Alizarin Red.

### Influence of the Pathological Conditions on SB: RT‐PCR, Western Blot, and Alizarin Red Evaluations

To assess the effect of pathological environment on Y201s fate, RT‐PCR, Western Blot, and Alizarin Red analyses were performed, as reported in Section 5.6.

For the gene expression analysis, *runx2, collIa2* (Hs01028956_m1)*, spp1* (Hs00959010_m1), and *vegf* (Hs00900055_m1) relative expression was evaluated and reported on day 1, day 7, and day 21, normalized to *gapdh*.

Western Blot protein analysis was performed on days 1, 7, 14, and 21 for RANKL (rabbit anti‐RANKL: 45 kDa; 2 µg mL^−1^, PA5‐110268, Invitrogen) and OPG (mouse anti‐OPG: 60 kDa, 1 µg mL^−1^, Ma5‐15960, Invitrogen), and the results were normalized to GAPDH and expressed respect to day 1.

Alizarin Red qualitative and quantitative analyses were performed on days 1, 7, 14, and 21.

### Influence of the Pathological Conditions on DL: Immunofluorescence, GAGs Quantification, and Stress Relaxation Analysis

Immunofluorescence Staining of Collagen X and Aggrecan: To observe the behavior of Y201‐Cs over 21 days of culture in terms of chondrogenic behavior and hypertrophy tendency, immunofluorescence staining was performed on DL‐Healthy (H) and DL‐Pathological (P) samples on days 1 and 21. After fixation using 4% PFA, permeabilization in 0.1% Triton‐X100, blocking with 1% bovine serum albumin (BSA), different dyes and antibody‐mediated labelings were applied: cell nuclei were stained with DAPI (1 µg mL^−1^ in PBS) for 5 min at RT, then samples were incubated for 2 h with Anti‐Collagen X monoclonal antibody (eBioscience) (10 µg mL^−1^ in 1% BSA) and Anti‐Aggrecan (ab3778 Abcam) (0.05 µg mL^−1^ in 1% BSA) according to manufacturer's instructions. After a wash in PBS, secondary antibodies solutions were added for 1 h: fluorescein‐labeled goat anti‐rabbit IgG (H + L) (F2765, Thermo Fisher Scientific) (1 µg mL^−1^ in PBS) for Anti‐Aggrecan and Goat Anti‐Mouse IgG (H&L) Alexa Fluor 594 (ab150116, Abcam) (2 µg mL^−1^ in PBS) for Anti‐Collagen X. Samples were washed and imaged with a Nikon A1R confocal microscope and analyzed with NIS Nikon software. ImageJ software (https://imagej.nih.gov/ij/) was used to count nuclei, Collagen X and Aggrecan positive cells, and data were expressed as the percentage of positive cells related to nuclei.

Glycosaminoglycans Quantification: Quantitative assessment of glycosaminoglycans (GAGs) production was performed on DL‐H and DL‐P with Alcian Blue (pH 2.5) staining on days 1, 14, and 21 of culture, as explained before.^[^ ^46^ ^]^ At each time point, DL‐H and DL‐P samples were fixed with 4% PFA, as explained before. Following, 500 µL of Alcian Blue solution were added to each sample for 30 min and then, samples were washed with dH_2_O until the blue disappeared. After, Guanidine hydrochloride was dissolved in dH_2_O (57% w/v) and 500 µL of the obtained Guanidine solution was added to each sample and let for 3 h under shaking. 100 µL were taken in triplicate from each well and reading was performed in absorbance at 630 nm with a FLUOStar Omega Plate reader (BMG LABTECH). Results were reported considering a calibration curve obtained from the bare Chondroitin 4‐sulfate sodium salt from bovine trachea in a range of 0–1 µg.

Stress–Relaxation Analysis: Stress–relaxation tests were performed as proposed by Scalzone et al.,^[^ ^54^ ^]^ using a single compression ramp at a speed of 10% min^−1^, until reaching the 10% strain. The strain was held constant for 800 s, while the load was recorded as a function of time. Peak Young's modulus (*E*_p_) was determined at 10% strain. The data obtained were analyzed using MATLAB R2021 software, by fitting a third‐order exponential decay to the relaxation curves. Obeying the generalized Maxwell model, two relaxation times were acquired: *τ*_1_ and *τ*_3_, which gave information on the poro‐viscoelastic behavior of healthy and pathological models after 14 days of culture, compared to day 1.^[^ ^55^ ^]^

### Statistical Analysis

The results' statistical significance was evaluated by GraphPad Prism Software (v. 8.4.1), using two‐way ANOVA with repeated measurements. Then, Tukey's post hoc test was carried out to highlight the main factors determining data variability. Statistical significance was set at *p < 0.05, **p < 0.01, ***p < 0.001, and ****p < 0.0001. All the experiments were performed in triplicates with at least three technical samples.

## Conflict of Interest

The authors declare no conflict of interest.

## Supporting information

Supporting Information

## Data Availability

The data that support the findings of this study are available from the corresponding author upon reasonable request.
