# Supplementary material for: An In Vitro Engineered Osteochondral Model as Tool to Study Osteoarthritis Environment
Source: Adv Healthc Mater. 2022 Oct 27;12(2):2202030. doi: 10.1002/adhm.202202030 (PMC11481676; doi:10.1002/adhm.202202030)
Supplement: Supplementary file 1 — Supporting Information [file ADHM-12-2202030-s001.pdf]

SUPPLEMENTARY DATA

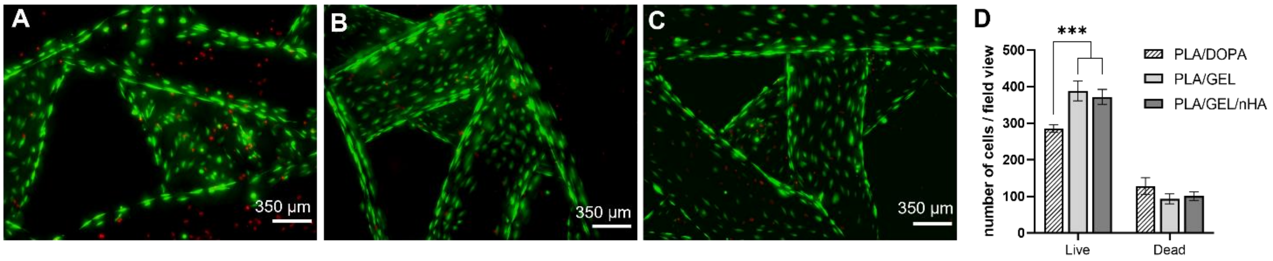

Figure S1: Y201 viability on PLA 3D printed scaffolds. Live/Dead viability assessment (live = green and dead= red) at day 3 on (A) PLA/DOPA, (B) PLA/GEL, (C) PLA/GEL/nHA. The histogram (D) represents the count for field of live and dead positive cells for PLA/DOPA, PLA/GEL and PLA/GEL/nHA. Scale bar: 350 µm. Statistics: ***p<0.001.
